# *In Vivo* Antiplasmodial Activity and Toxicological Analyses of the Ethanolic Leaf and Twig Extract of *Faurea speciosa* Welw. (Proteaceae)

**DOI:** 10.1155/2021/7347532

**Published:** 2021-08-29

**Authors:** Felix Ayisi, Caleb Nketia Mensah, Lawrence Sheringham Borquaye

**Affiliations:** ^1^Department of Chemistry, Kwame Nkrumah University of Science and Technology, Kumasi, Ghana; ^2^Central Laboratory, Kwame Nkrumah University of Science and Technology, Kumasi, Ghana

## Abstract

In Africa, medicinal plants are commonly used to treat malaria and other diseased conditions. The ethanolic leaf and twig extract of *Faurea speciosa* has been shown to possess promising antiplasmodial properties. This present study was aimed at investigating its antiplasmodial effect *in vivo*. Qualitative phytochemical screening was carried out on the plant samples using standard methods. The antiplasmodial effect against early infection, curative effect against established infection, and prophylactic effect against residual infection were studied *in vivo* in *Plasmodium berghei*-infected mice while the carrageenan-induced edema model in chicks was used for anti-inflammatory tests. The phosphomolybdenum and DPPH radical scavenging assays were used in the evaluation of antioxidant potential. Acute toxicity of the extract was evaluated using the Organization for Economic Cooperation and Development (OECD) guidelines. Phytochemical screening of plant samples revealed the presence of flavonoids, coumarins, tannins, saponins, and glycosides. *Faurea speciosa* leaf and twig extract exhibited significant antiplasmodial activities in the mouse model with parasite suppression rates of 66.63%, 71.70%, and 56.93% in the suppressive, curative, and prophylactic tests, respectively. A 55.50% reduction of edema in the anti-inflammatory test indicated moderate success in reducing inflammation. The total antioxidant capacity of the extract was determined to be 65.4 mg AAE/g of extract, while in the DPPH radical scavenging assay, the IC_50_ value was found to be 499.4 *μ*g/mL. With the exception of an inconsistent rise in urea level, there was no significant difference in the other biochemistry parameters in the acute toxicity studied. The median lethal dose (LD_50_) of the extract was over 2000 mg/kg. The results of this study show that *Faurea speciosa* leaf and twig extract has promising antimalarial capabilities and is fairly safe at low concentrations.

## 1. Introduction

Malaria is one of the world's deadliest infectious diseases. It is caused by a protozoan parasite of the genus *Plasmodium* and contributes substantially to mortality and morbidity in many developing countries. According to the World Malaria Report for 2020, 229 million new cases of malaria and 409,000 malaria deaths worldwide were registered with a 95% mortality rate for 31 African countries [1]. In Ghana, malaria is a public health hazard and is among the foremost causes of death in pregnant women and children under 5 years old. Most people who become sick cannot afford orthodox drugs, and so they resort to medicinal plants for the treatment of malaria [2]. Available evidence from history suggests parasite resistance to current therapeutics is inevitable. Resistance to quinine, the first documented antimalarial drug, was recorded in the 1910. Chloroquine-resistant parasites were detected in the 1950s whereas pyrimethamine-resistant *Plasmodium* parasites were observed the same year the drug was introduced onto the market [3]. Reports of the existence of resistant parasite strains to the currently used artemisinin and its derivatives present a clear and formidable challenge [4]. This has necessitated a need to uncover new lead compounds for malaria drug discovery programs. Medicinal plants have been promising targets for the search of new antimalarial agents as most of them are rich in secondary active metabolites [5]. The most efficient antiplasmodial drugs have come from plant metabolites as evidenced by two of the most successful antimalarial drugs—quinine and artemisinin [6].

During malaria infection, the *Plasmodium* parasite feeds on the host's haemoglobin as a source of amino acids and also to regulate osmotic pressure which is necessary for its growth [7]. The feeding process leads to the production of reactive oxygen species (ROS) [8]. The widely studied and understood ROS include the superoxide anion (O_2_^−2^), hydroxyl radical (OH^−^), hydrogen peroxide (H_2_O_2_), and hypochlorous acid (HOCl). At elevated levels, ROS can cause severe damage to host DNA and oxidize cell membrane protein and lipids [9]. Imbalance between ROS and the host's antioxidative systems leads to oxidative stress, which could lead to cell damage and eventual death. Antioxidants can neutralize ROS and reactive nitrogen species (RNS) and decrease oxidative stress while restoring oxidative balance. The use of antioxidants in malaria therapy in order to lessen oxidative stress-related complications in the host has been explored [10]. It has been shown that extracellular ROS can induce inflammation in malaria since the ROS are produced in excess during infection [11]. The presence of ROS in higher levels can damage cells and hence the body's immune response which usually leads to inflammation. Inflammation is one mechanism used by the body to fight against infections, toxins, and injuries, in an attempt to heal itself. When the inflammatory response is short term, it serves a useful purpose by protecting the body against further damage and aids recovery [12]. In malaria infection, inflammatory response by the host plays a key role in the disease pathology [11]. Arresting inflammation and triggers of this inflammatory response—such as ROS and RNS—could potentially ameliorate the harmful effects of the disease.

For centuries, plants have been used traditionally as a source of cure for various ailments. In recent times, some adverse effects have been observed with the use of some medicinal plants. Medicinal plants have been patronised widely but with little or no scientific evidence on their efficacy and safety. Therapeutic phytoconstituents are largely regarded as safe and nonpoisonous because they are “natural,” but they could be potentially toxic [13]. Some medicinal plants including *Lantana camara*, *Pteleopsis hylodendron*, *Morinda lucida*, *Tetrapleura tetraptera*, *Khaya senegalensis* (Desv) *A. Juss*, and *Jatropha tanjorensis* contain potentially hepatotoxic constituents [14, 15]; hence, toxicity assessment of plants with proven therapeutic use is of utmost important. Herbal medicines are commonly used to treat malaria infections in Ghana [16, 17]. In light of the knowledge that some plants maybe potentially toxic at therapeutic concentrations, it is imperative to evaluate commonly used and potential medicinal plants for their toxicity.

*Faurea speciosa* (*synonym: Faurea rochetiana*) belongs to the family Proteaceae, and it is mostly found in the West African subregion. Ethnobotanically, the leaf and root are used to cure ear ailments [18]. *In vitro* studies have shown that *Faurea speciosa* has a moderate antimalarial activity and contains alkaloids, tannins, and coumarins [19]. No study on whether the plant also has *in vivo* activity exists in literature. This research therefore examined the plant's antiplasmodial function in a rodent malaria model and also assessed its anti-inflammatory and antioxidant activities. Toxicological assessment of the plant was also carried out. Antiplasmodial assessment was performed in a murine model using *Plasmodium berghei* (*ANKA strain*) for infection. To evaluate the anti-inflammatory action, the carrageen-induced foot edema model in chicks was employed. The antioxidant activities were conducted using the phosphomolybdenum and 2,2-diphenyl-1-picrylhydrazyl (DPPH) radical scavenging assays. Acute toxicity and liver and kidney function tests were used in safety evaluations.

## 2. Experimental Methods

### 2.1. Chemicals

The chemicals used throughout the study were of analytical grade. Ethanol (99% anhydrous grade) and 2,2-diphenyl-1-picrylhydrazyl (DPPH, purity 95%) were obtained from Sigma-Aldrich (St. Louis, USA). Artesunate and mefloquine were obtained as pure powders (99%) from Ernest Chemists Limited (Tema, Ghana). Milli-Q water was used in the preparation of all calibration standards and other reagents.

### 2.2. Plant Material Collection and Processing

Plant materials were collected from Adawso (5.9489°N, 0.2127 W) in the Eastern Region of Ghana, in January of 2020. The plant sample was authenticated at the Department of Herbal Medicine-KNUST, where the herbarium specimen has also been kept (KNUST/HMI/2020/L/006). The plant material was then cleaned by removing any debris. The twigs were chopped into smaller pieces, then air dried together with the leaves for about 21 days, and then milled together into a course powder. The powdered plant material (600 g) was extracted by Soxhlet with absolute ethanol as the solvent. Ethanol was chosen as the solvent because of the manner in which the plant is traditionally prepared for use in Ghanaian folkloric medicine. The collected ethanol extract was vacuum concentrated (Cole Parmer Rotary Evaporator N-1110, China) and evaporated to complete dryness on a water bath, yielding a sticky dark green extract. This was kept at 4°C for storage until further use.

### 2.3. Phytochemical Screening

The *Faurea speciosa* ethanol extract was examined for the presence of phytochemicals such as alkaloids, saponins, triterpenoids, tannins, sterols, flavonoids, coumarins, and glycosides using standard procedures for qualitative screening of phytochemicals. To test for tannins, 3 mL of 10% lead acetate solution was added to 3 mL of plant extract. A bulky white precipitate, when present, is an indication of the presence of tannins. In testing for steroids, 5 mL of the extract was remade in CHCl_3_. Concentrated H_2_SO_4_ was added carefully to the side of the test tube to form a lower layer. A reddish-brown/cherry red color at the interface is an indication of the presence of a steroid with a triterpenoid nucleus. In testing for flavonoids, a strip of filter paper was dipped into the liquid extract and then dried. The filter paper was exposed to an NH_3_ solution, which was then followed by fumes of concentrated HCl. An intense yellow coloration upon exposure to the fumes of concentrated NH_3_ solution and subsequent disappearance of the color upon exposure to the fumes of concentrated HCl indicated a positive flavonoids test. In the test for alkaloids, extract was dissolved in ammoniacal alcohol, and resulting solution was filtered and then evaporated to dryness. The residue was reextracted with 1% H_2_SO_4_, filtered, and the filtrate basified with NH_3_(aq). It was then shaken with CHCl_3_; the chloroform layer was then separated and concentrated to dryness. The residue was dissolved in 1% H_2_SO_4_. Formation of an orange-to-red precipitate after the addition of Dragendorff's reagent showed a positive test for alkaloids. In testing for coumarins, plant extract was dissolved in 5 mL of diethyl ether, followed by evaporation to complete dryness. The residue was then redissolved in hot water. Following a cooling period, 10% ammoniacal solution (0.5 mL) was added to a small part of the dissolved residue. The presence of an intense blue-to-green fluorescence under UV light indicated a positive coumarin test. To test for the presence of glycosides, plant extract was dissolved in dilute HCl (5 mL). This was followed by heating for 2 minutes on a water bath. The warm solution was thereafter filtered and basified with 20% NH_4_OH. Fehling's A and B solution (1 mL) was then added to the filtrate and again heated for 2 minutes. The presence of a brick-red precipitate showed that glycosides were present [20].

### 2.4. *In Vivo* Antiplasmodial Assays

*(1) Experimental Animals and Parasite*. Balb/c albino mice aged between 6 and 8 weeks and 23 to 30 g were obtained from the Animal Unit of the Centre for Plant Medicines Research (CPMR). Mice were housed in cages at a temperature of 27°C with a day/night cycle of 12 hours each. There was always food and water available for the animals. *Plasmodium berghei* (ANKA strain) was obtained from the Department of Pharmacology-KNUST. Serial blood transfer from infected mice to noninfected mice was done per week to maintain the parasites. All assays used were based on the methods used by Nardos and Makonnen [21] and Ayisi and coworkers [16].

*(2) Parasite Inoculation*. Each mouse used was injected intraperitoneally with infected blood (0.2 mL) containing approximately 1 × 10^7^*P. berghei* parasites. This was prepared by sacrificing donor mice and blood collected by cardiac puncture into heparinized vacutainer tube containing 0.5% trisodium citrate. The blood was diluted with isotonic saline in proportions according to the parasitemia levels (20-30%).

#### 2.4.1. Suppressive Test

*Plasmodium berghei-*infected Balb/c mice were put in 5 groups of three each based on body weight. On the first day (D0), mice in all groups were inoculated with *P. berghei*. Three hours after inoculation, treatment for animals in the various groups begun. Mice in groups 1, 2, and 3 received 100, 200, and 400 mg/kg of *Faurea speciosa* extract, respectively, while group 4 received 4 mg/kg of artesunate (positive control) and 10 mL/kg of phosphate-buffered saline (PBS) at a pH of 7.4 was given to group 5 (negative control) for 4 continuous days (D0–D3) between 8 am and 9 am. On day five (D4), Giemsa-stained thin blood film was made from blood drawn from the tail of each mouse. Duplicates of each thin film were made. The total number of red blood cells (RBCs) and infected RBCs was counted [21]. Parasitemia was obtained from

(1) $$\begin{array}{c} \%\text{Parasitemia}=\frac{\text{number of infected RBCs}}{\text{Total number of RBCs}}\times 100. \end{array}$$

For each sample, chemosuppression was derived from

(2) $$\begin{array}{c} \%\text{Chemosuppresion}=\frac{\left(A-B\right)}{A}\times 100, \end{array}$$

where “*A*” represents average parasitemia of the negative control group and “*B*” the parasitemia of the test group.

The mean survival time (MST) was established over a period of 30 days for each group using

(3) $$\begin{array}{c} \text{MST}=\frac{\text{sum of survival time of all mice in group }\left(\text{days}\right)}{\text{total number of mice in that group}}. \end{array}$$

#### 2.4.2. Curative Test

On the first day (day 0), fifteen mice were inoculated intraperitoneally with standard inocula of 1 × 10^7^*P. berghei-*infected erythrocytes. Seventy-two hours later (day 3), the mice were placed into 5 groups of 3 mice each, based on body weight. Groups 1, 2, and 3 received 100, 200, and 400 mg/kg extract, respectively, each day, and group 4 received PBS (10 mL/kg) while Group 5 also received artesunate (4 mg/kg) daily. All extracts, PBS, or standard drugs were administered orally for 5 days continuous. Thin films were made with blood obtained from the tail of each mouse and fixed with methanol, stained with Giemsa in duplicates, and parasitemia determined microscopically on day 3 and day 7. Parasitemia and chemosuppression were derived from equations (1) and (2), respectively. The mean survival time for each group was obtained from equation (3).

#### 2.4.3. Prophylactic or Repository Test

Test mice were put into 5 groups of 3 mice each based on their body weight. By oral administration, groups 1, 2, and 3 were given 100, 200, and 400 mg/kg/day of the extract, respectively; groups 4 and 5, respectively, received 4 mg/kg/day of mefloquine (positive control) and 10 mL/kg of PBS (negative control). The treatment lasted for three consecutive days after extract/drug administration (D0–D2). The mice were then inoculated with *P. berghei* on day four (D3). After 72 hours of care, duplicate Giemsa-stained thin spray samples from tail blood were prepared to test the extent of parasitemia. Parasitemia and chemosuppression were derived from equations (1) and (2), respectively. The mean survival time for each group was obtained from equation (3).

### 2.5. Antioxidant Activities

#### 2.5.1. DPPH Method

The radical scavenging action of the ethanol extract was measured by the DPPH (1,1-diphenyl-2 picrylhydrazyl) assay. A small amount of methanolic DPPH reagent (0.1 mM; 100 *μ*L) was added to 100 *μ*L of varying concentrations of *Faurea speciosa* extract and ascorbic acid (which was used as standard drug). A solution of equal volumes of methanol and DPPH served as a control mixture. Resulting solutions were vigorously mixed and kept in a dark room for thirty minutes at ambient temperature. The absorbance of each mixture was measured at 517 nm (PerkinElmer Lambda 35). The difference in absorbance between the test and the control samples was calculated and expressed as % scavenging of DPPH radical using

(4) $$\begin{array}{c} \%\text{Scavenging Effect}=\frac{A_{\text{c}}-A_{\text{s}}}{A_{\text{c}}\;}\times 100, \end{array}$$

where *A*_s_ is the absorbance of extract/drug mixture and *A*_c_ is the absorbance of the blank control.

The IC_50_ value, defined as the concentration of extract or drug required to scavenge 50% of the DPPH, was derived from a graph of % scavenging effect versus extract or drug concentration [22, 23].

#### 2.5.2. Total Antioxidant Capacity Assay

The test is based on the reduction of molybdenum (Mo^+6^ to Mo^+5^) and subsequent formation of a green phosphate molybdate (Mo^+5^) complex at acidic pH. One millilitre of various concentrations of *Faurea speciosa* extract or standard ascorbic acid was added to tubes containing 3 mL of the reagent solution (28 mM Na_2_HPO_4_, 0.6 M H_2_SO_4_, and 4 mM (NH_4_)_6_Mo_7_O_24_) and incubated at 95°C for 90 minutes. The mixture was allowed to cool to room temperature and absorbance determined at 695 nm (PerkinElmer Lambda 35). A blank solution containing all other solutions with the exception of extract or standard drug was treated as earlier described. Each experiment was conducted 3 times, and the total antioxidant capacity was expressed as equivalents of ascorbic acid [22, 24].

### 2.6. Anti-Inflammatory Activity

#### 2.6.1. Animals

One-day old chicks (Gallus; strain shaver 579) were used in this study. The chickens were kept in 34 × 57 × 30 cm^3^ stainless steel cages with 15 chicks in each cage with a 12-hour day/light cycle at the Animal House of the Department of Pharmacology, KNUST. Feed and water were made available at all times in feeders and water troughs. The chicks were then used for the study after 7 days of acclimatization.

#### 2.6.2. Carrageenan-Induced Foot Edema in Chicks

The carrageenan foot edema model was used to evaluate the anti-inflammatory properties of *Faurea speciosa* in chicks. Seven-day posthatched chicks (45-60 g) were divided into 7 different groups, with each group having 5 chicks (*n* = 5). The foot volumes of each chick were measured with a Vernier calliper. Carrageenan (0.1 mL, prepared as 1% *w*/*v* in normal saline) was administered subplantar into the right footpads of the chicks. The foot volumes were again measured an hour after the carrageenan administration. Extracts were administered orally to chicks in groups 1 to 4 at 10, 30, 100, and 300 mg/kg, respectively. Groups 5 and 6 received 0.3 mg/kg dexamethasone and diclofenac, respectively, via intraperitoneal injection, and then, group 7 (control group) was instead given normal saline. Foot volumes were then measured hourly for the next 6 hours.

#### 2.6.3. Analysis

The difference in foot volumes before carrageenan administration and at the various time intervals after the administration was quantified to evaluate the edema component of inflammation. Foot volumes were calculated as percentage of change from their values at time zero and then averaged for each treatment group. The change in foot volume, expressed as a percentage, was derived with

(5) $$\begin{array}{c} \%\text{change in foot volume}=\frac{F_{t}-F_{\text{o}}}{F_{\text{o}}}\times 100, \end{array}$$

where *F*_*t*_ is the foot volume at time, *t*, posttreatment and *F*_o_ is the initial foot volume measured at time = 0 before carrageenan administration.

Total foot volume was measured in the subjective unit for any treatment, and the edema inhibition percentage for treatment groups was estimated as a region under the curve (AUC) using

(6) $$\begin{array}{c} \%\text{Inhibition of edema}=\frac{\text{AUC control}-\text{AUC treatment}}{\text{AUC control }}\times 100. \end{array}$$

In order to analyze the variations in all data sets, a one-way analysis of the variance (ANOVA) and Dunnett *post hoc* test were used. The GraphPad Prism version 6.0 (GraphPad Software, San Diego, CA) and Microsoft Excel were used in data analysis [25, 26].

### 2.7. Safety Evaluation

#### 2.7.1. Acute Toxicity

The acute toxicity was evaluated using the Organization for Economic Cooperation and Development (OECD) guidelines for testing of chemicals [27]. The rodents used in this test were female Wistar albino rats that weighed between 125 and 170 g and were about 8-10 weeks old. The animals were divided into 4 groups of 5 animals each. The animals were kept in cages for 7 days to acclimatize with the laboratory conditions before experiments were carried out. The Wistar albino rats were denied food overnight before commencement of experiments; however, there was water available. Normal saline was administered to animal in group 1, which was the control group, while animals in groups 2, 3, and 4 were given 50 mg/kg, 300 mg/kg, and 2000 mg/kg doses of *Faurea speciosa* extract, respectively. The animals received feed and water as often as necessary throughout the experiment. All animals were checked 30 min, 60 min, and 4 hours after extract and vehicle administration, and this monitoring continued every day for the next 14 days for death detection and any comportment, autonomous, or neurological changes. Feed and water consumption was also monitored. Body weights were recorded on days 1, 7, and 15. On the 15^th^ day, the animals were anesthetized and 2-3 mL blood samples were collected by cardiac puncture into small tubes that contained no anticoagulants. Collected samples were used in blood chemistry analysis.

#### 2.7.2. Liver and Kidney Function Tests

Collected blood samples were used in monitoring hepatic and renal function. The key parameters studied included urea and creatinine for kidney function and then alanine transaminase (ALT), alkaline phosphatase (ALP), aspartate transaminase (AST), total bilirubin (TB), indirect bilirubin (IB), and direct bilirubin (DB) for liver function. An automated analyzer (Automated Biochemical Analyzer) was employed in blood chemistry analyses using standard protocols earlier reported [20].

### 2.8. Statistical Analysis

Values were expressed as mean ± SEM. The one-way ANOVA was used for analysis at 95% confidence level comparison of the findings. Values of *p* < 0.05 were considered significant.

## 3. Results

### 3.1. Extraction and Phytochemical Tests

The extraction of 600 g *Faurea speciosa* sample with ethanol gave a yield of 14.5%. The extract was a dark, green sticky paste. The results of the phytochemical screening showed that phytoconstituents such as saponins, tannins, flavonoids, coumarins, and glycosides were present (Table 1). Alkaloids, triterpenoids, and sterols were however not detected under the experimental conditions.

### 3.2. Antiplasmodial Activity

#### 3.2.1. Peter's Four-Day Suppressive Test

In evaluating the schizontocidal activity of the ethanolic extract of *F. speciosa* against mice infected with *P. berghei*, the 4-day suppressive test was employed. The extract showed a dose-dependent suppressive effect on the parasites. These effects, at all doses tested, were statistically significant relative to the negative control (*p* < 0.001). The parasitemia level of the extract-treated groups ranged from 31 to 77% whereas that of the vehicle-treated group was 93.72%. Suppression of parasitemia was 17.55, 37.08, and 66.63% for 100, 200, and 400 mg/kg of extract, respectively. In comparison to artesunate, the effect of the extract was moderate (Table 2). At all doses of the extract, there was a significant increase in the MST of the animals (14-21 days) relative to that of the vehicle-treated group (8.80 days). An MST of 29.20 days was recorded for the artesunate-treated group, which was higher than the MST for any of the extract-treated groups (Table 2).

Malaria infection is often associated with body weight loss, but the standard drug (artesunate 4.0 mg/kg) as well as the extract at all administered doses, 100, 200, and 400 mg/kg, prevented malaria-associated body weight loss in *P. berghei*-infected mice. A slight decrease in body weight of the animals was recorded in the vehicle-treated group (Table 3). The vehicle-treated group recorded an average body weight loss of 1.42%. For the extract-treated groups, 4.21%, 5.08%, and 1.09% gains in body weight were observed in animals given 100, 200, and 400 mg/kg extracts, respectively. All extract doses, except 400 mg/kg, were better compared to the artesunate-treated group which recorded a gain in body weight of 4.02%. Slight decrease in rectal temperatures was observed for the vehicle- and extract-treated groups. The extract was unsuccessful in preventing reduction in the body temperature of the mice. In the artesunate-treated group, the rectal temperature of the mice was maintained throughout the duration of the experiment. No significant difference in the rectal temperature of mice was observed in all the various treatment groups (Table 3).

#### 3.2.2. Curative Test

The curative test evaluates a drug's capability to eradicate parasites at an established stage of a disease. The extract produced a dose-dependent decrease in parasite counts on both days 3 and 7 (Table 4). The mean parasitemia for the extract- and artesunate-treated groups was much lower on day 7 when compared to day 3. On day 7, the mean parasitemia was 41.95, 22.79, and 21.83%, for 100, 200, and 400 mg/kg treatment groups, respectively. Administration of 4 mg/kg artesunate reduced the mean parasitemia to 5.33% on day 7. For the vehicle treatment group, parasitemia levels increased from 66.17 on day 3 to 77.14% on day 7. The percent chemosuppression on day 7 was <50% for the 100 mg/kg treatment group but ≥70% for the groups that received 200 mg/kg and 400 mg/kg of extract. The artesunate-treated group showed a 93.09% chemosuppression on day 7 (Table 4). The extract dosage also influenced the mice's survival time. Mice administered with 100 and 200 mg extract/kg survived for 13 and 16 days, respectively, but those administered with 400 mg/kg survived for 20 days. Mice treated with artesunate survived 28 days after administration which was significantly (*p* < 0.0001) better than the vehicle- or extract-treated groups (Table 4). A gain in body weight was observed from dgteconnect.spi-global.comday 3 to day 7 for all animals in extract- and artesunate-treated groups (Table 5). However, animals in the vehicle-treated group showed a slight decrease in average weight (-2.88%) from day 3 to day 7. A marginal increase in rectal temperature of 0.17°C was observed in the vehicle-treated group. On the other hand, the extract- and artesunate-treated groups recorded a drop in rectal temperature from day 3 to day 7. No significant change in body temperature was detected between the vehicle-, artesunate-, and extract-treated groups for the duration of the experiment (Table 5).

#### 3.2.3. Prophylactic Test (Repository Test)

Prophylactic test assesses a medication's preventive ability. There was a significant (*p* ≤ 0.05) and dose-dependent decrease in parasite count in the extract-treated group. The mean percentage chemosuppression was 47.82, 47.86, and 56.93 at 100, 200, and 400 mg extract/kg, respectively, while mefloquine at 4 mg/kg produced an 83.82% chemosuppression. The parasite level also decreased substantially with increase in the dose of extract when compared with negative control. The mean percent parasitemia at 100, 200, and 400 mg extract/kg was 31.90, 31.88, and 26.33, compared with 61.14 for the negative control group. However, the mefloquine-administered group recorded mean parasitemia of 9.90% (Table 6). The mice in the negative control and 100 mg/kg extract-treated groups observed significant reduction in body weight by 6.83% and 1.21%, respectively. However, the 200 mg/kg-, 400 mg/kg-, and mefloquine-treated groups recorded an increase in body weight. Rectal temperature reduced in groups treated with 100 mg/kg body weight, mefloquine, and the negative control. Meanwhile, groups treated with 200 and 400 mg/kg body weight recorded increased rectal temperature of 0.17°C and 0.33°C, respectively (Table 7).

### 3.3. Antioxidant Activity

The phosphomolybdenum (PM) and DPPH assays were used to assess the antioxidant capabilities of the ethanolic leaf and twig extract of *Faurea speciosa*, and the data is presented in Table 8. The total antioxidant capacity (TAC) attained from the PM assay was 65.4 ± 0.5 mg/100 g AAE. The IC_50_ value of *Faurea speciosa* in the DPPH radical scavenging assay was estimated to be 499.7 ± 5.8 *μ*g/mL. Comparatively, 16.3 ± 0.5 *μ*g/mL of the ascorbic acid was required in scavenging 50% of DPPH radicals.

### 3.4. Anti-Inflammatory Activity

In the anti-inflammatory assay, *Faurea speciosa* extracts showed significant anti-inflammatory activity (*p* < 0.001) when compared to the standard drugs. Injection of carrageenan into the paw of chicks induced a progressive edema reaching its maximum at the 90^th^ minute for the extract-treated groups but continued till the 2^nd^ hour for the group that received only vehicle. After extract, diclofenac, or dexamethasone administration, a sharp reduction in foot volume was observed till the 6^th^ hour. Maximal foot edema was recorded which was achieved at the 2^nd^ hour for the vehicle-treated group. A dose-dependent relationship was observed for the *Faurea speciosa* extract groups, and the same was evident in the percent inhibition of edema that was calculated for the extract, 41.01, 49.96, and 55.50 percent inhibitions of edema observed for 10, 30, and 100 mg/kg treatment groups, respectively (Table 9). For groups that received diclofenac and dexamethasone, percent inhibitions were calculated to be 47.61% and 60.03%, respectively.

### 3.5. Acute Toxicity and Behavioural Changes

Wistar female albino rats were administered 50, 300, and 2000 mg/kg of *Faurea speciosa* extracts in evaluating the acute toxicity of the extract. A control group received only the vehicle (saline), and all treatment groups were then studied for 14 days. Throughout the study period, all behavioural and morphological characteristics were seen to be normal. No deaths, tremors, diarrhoea, convulsion, or salivation signs were observed in the rats in any of the treatment or control groups. Thus, there was no disparity between treatment groups and control in clinical findings. During the study, a gradual increase in the body weight of the rats was observed. For the control group, there was about 28% increase in body weight during the experimental period. For the treatment groups, the increase in body weight was 10.61, 8.54, and 16.12%, respectively, for groups that received 50, 300, and 2000 mg/kg of extract (Table 10). There was no significant change (*p* > 0.05) in body weight in animals in the 50 mg/kg treatment group relative to the control. However, a significant (*p* < 0.001) weight increase was observed in the 300 and 2000 mg/kg treatment groups. The lethal dose (LD_50_) was estimated to be more than 2000 mg/kg.

**Figure 1:**
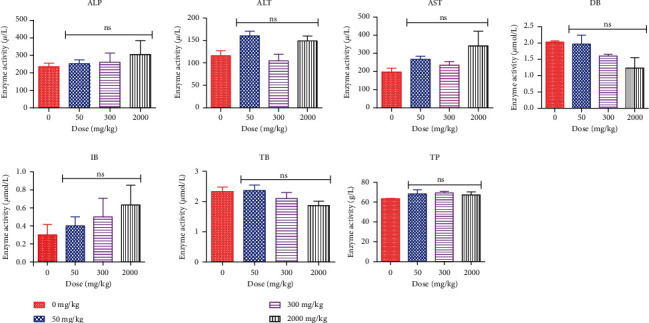
Liver function test: effect of ethanolic extract of *Faurea speciosa* on important biomarkers of liver function. ALT: alanine aminotransferase; AST: aspartate aminotransferase; ALP: alkaline phosphatase; DB: direct bilirubin; IB: indirect bilirubin; TB: total bilirubin; TP: total protein. Each bar represents mean ± SD for 3 replicate experiments (*n* = 3). *p* < 0.05 was considered to be statistically significant; ns: not significant (*p* > 0.05) (one-way analysis of variance (ANOVA) followed by Dunnett's post hoc test).

**Figure 2:**
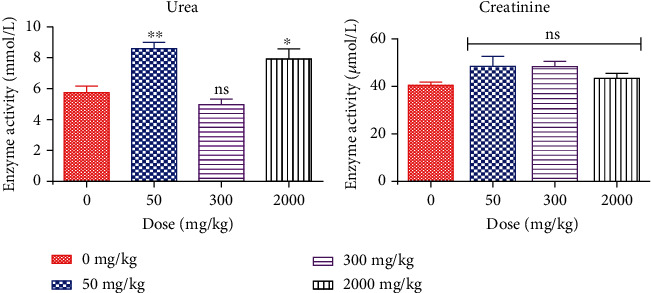
Kidney function test: effect of ethanolic extract of *Faurea speciosa* on important biomarkers of kidney function. Each bar represents mean ± SD for 3 replicate experiments (*n* = 3). *p* < 0.05 was considered to be statistically significant; ns: not significant (*p* > 0.05); ^∗/∗∗^significant (*p* < 0.05) (one-way analysis of variance (ANOVA) followed by Dunnett's *post hoc* test).

To assess the effect of *Faurea speciosa* extract on hepatic (Figure 1) and renal (Figure 2) function, main enzymatic activities related to the proper functioning of the liver and kidneys were determined. The levels of urea and creatinine were used as a marker for kidney function whereas the activities of alanine transaminase (ALT), alkaline phosphatase (ALP), aspartate transaminase (AST), total bilirubin (TB), indirect bilirubin (IB), and direct bilirubin (DB) were used to evaluate liver function. The average urea level in the 50 and 2000 mg/kg treatment groups was about 49% and 38%, respectively, higher than that of the control group and this represented a significant (*p* < 0.05) increase in urea level (Figure 2). For all other parameters measured, however, no significant differences existed between values obtained for the treatment groups relative to those obtained for the control group (*p* > 0.05).

## 4. Discussion

This study sought to evaluate the *in vivo* efficacy and safety of *Faurea speciosa* leaf and twig extract that is commonly used in Ghanaian herbal medicine for the treatment of malaria. Initial phytochemical screening indicated that coumarins, flavonoids, glycosides, tannins, and saponins were present in the ethanolic extract of the leaves and twigs of the plant (Table 1). A previous work done on the crude extract of *Faurea speciosa* leaves and twigs showed the presence of alkaloids, tannins, and coumarins [19]. Extract from the previous work was obtained via cold maceration while this current study used Soxhlet. Plant samples for this study were collected in January while the earlier researchers collected theirs in August, both from different geographical locations. The difference in extraction techniques, time of sampling, and sampling locations might account for the disparities in the phytochemicals reported. Phytochemicals like alkaloids, terpenes, flavonoids, coumarins, and limonoids have been acknowledged as possible antimalarial agents. These phytochemicals may function by themselves individually or in collaboration with others in order to demonstrate the biological activities observed [28].

When infected with *P. berghei*, rodent models produce disease features similar to those of human plasmodial infection [29]. The *in vivo* results from this work indicated that the ethanol extract from *Faurea speciosa* leaves and twigs has substantial suppressive, curative, and prophylactic effects against the parasite at safe doses. This confirms an earlier report which revealed that crude extract from *Faurea speciosa* leaves and twigs showed promising antiplasmodial effect *in vitro* [19]. The extract exhibited substantial dose-dependent chemosuppression in all the test groups in all 3 assays used. The standard drug for suppressive and curative tests, artesunate, administered at 4 mg/kg body weight, recorded a chemosuppression of about 93%. The *Faurea speciosa* extract exhibited a much better antiplasmodial effect in the curative test than in the suppressive test. A chemosuppression of parasitemia at the highest dose (400 mg/kg) was 71.70% and 66.63% for curative and suppressive tests, respectively. In the prophylactic test, the extract had a chemosuppression of 56.93% at the highest dose of 400 mg/kg, while the standard drug mefloquine (4 mg/kg) recorded a chemosuppression of 83.82%. The precise mode of action of the *Faurea speciosa* extract is not clarified; however, antiplasmodial properties of plant products have proven to be dependent on their active phytoconstituents [21]. The antiplasmodial properties exhibited by the ethanolic leaf and twig extract of *Faurea speciosa* may consequently be a result of the phytoconstituents acting individually or synergistically through a combination of actions. Indeed, many antimalarial drugs function by a multitude of mechanisms. Artemisinin, for example, is proposed to function via three routes against the malaria parasite. Artemisinin interacts with *Pf*ATP6 and exerts irreversible damage to the protein. It has also been suggested that artemisinin plays a role in the inhibition of hemozoin formation. Finally, the parasite mitochondria activate artemisinin, which induces production of free radicals to damage important molecules in the parasite [30].

Anaemia, a decrease in body weight, and an elevated temperature are characteristics of malaria infection in animals [31]. Any effective antiplasmodial agent should therefore be able to avoid body weight loss and maintain body temperature during a malaria treatment regime. The *Faurea speciosa* extract in all three test cases was able to prevent weight loss, except for the 100 mg/kg treatment group in the prophylactic test where a 1.21% weight loss was recorded. All the saline-treated groups (negative control) in the suppressive, curative, and prophylactic tests recorded a percent weight loss of 1.42, 2.88, and 6.83, respectively. Among the symptoms of malaria infection in humans, body temperature is elevated. In terms of body temperature, the mice in the negative control groups in both suppressive and curative tests suffered a general decrease in body temperature throughout the test period. In the suppressive and curative tests, the infected mice developed slight drops in rectal temperature by a maximum of 0.53°C. This prolonged lowered temperature in mice could be associated with the effect of the malaria parasite on the host which may result in body heat loss and eventually death of mice [32]. It has also been reported that malaria parasites also affect host carbohydrate, lipid, and protein metabolisms. Additionally, a reduction in metabolic rate of *P. berghei*-infected host coupled with a reduction in internal body temperature has been cited [33]. Consequently, active antimalarial potentials are expected to avert the decrease in rectal temperature. Thus, *Faurea speciosa* crude extract prevented the drop in rectal temperature of mice contrary to the control group. In standard screening tests, antimalarial agents with >30% suppressive effect on parasitemia and can extend the survival date of treated mice relative to the control group mice are mostly classified as effective in standard screening tests [34]. In this current *in vivo* antimalarial assessment, the suppressive, curative, and prophylactic test results show that *Faurea speciosa* is active in treating malaria.

Due to the overproduction of reactive species—ROS and RNS—by the Plasmodium parasite during malaria infection, oxidative stress in host cells is usually observed [11]. When left uncontrolled, oxidative stress could damage proteins, nucleic acids, and lipids and thus lead to severe tissue injury. Thus, plant extracts that could scavenge RNS and ROS in addition to their antiplasmodial capabilities could be very useful. In lieu of this, the antioxidant activity of the extract was assessed. The capacity of *Faurea speciosa* extract to scavenge free radicals was studied in the DPPH assay, where the IC_50_ value of the extract was determined to be 499.4 ± 5.78 *μ*g/mL. That of the standard ascorbic acid was 16.34 ± 0.055 *μ*g/mL. In the phosphomolybdenum (PM) assay, a TAC of 6.54 ± 0.53 g AAE/100 g was recorded. Both results point to a moderate antioxidant effect by the *Faurea speciosa* extract. In another similar study, the aqueous extract of *Haematostaphis barteri* was shown to possess good curative and prophylactic antiplasmodial activity but a relatively lower total antioxidant capacity (42 mg AAE/g of extract) [35]. RNS and ROS have been implicated in the inflammation cascade. Since malaria is an inflammatory-driven cytokine disease and results in the release of various inflammatory mediators [11], the extract of *Faurea speciosa* was also investigated for its ability to reduce inflammation. The ethanolic extract displayed moderate anti-inflammatory effect. A dose-dependent reduction in edema was observed. At the maximum dose of 100 mg/kg, a 55.89% reduction in edema was observed. Taken together, the results of the antioxidant and anti-inflammatory tests suggest that extracts of *Faurea speciosa* could scavenge and neutralize reactive species that may lead to oxidative stress and induce inflammation. Additionally, the extracts could potentially interfere with the production and/or release of inflammatory mediators and thus exert an anti-inflammatory action. These, coupled with the *in vitro* and *in vivo* antiplasmodial activity of the extract, suggest a basis for its use in folkloric medicine for the treatment of malaria and malaria-like symptoms.

Safety evaluation of the *Faurea speciosa* extract was undertaken to ascertain its effect on the normal growth pattern of rats. At the end of 14-day observation after extract administration, the body weight of all animals in the treatment and control groups increased progressively throughout the period of the study (Table 10). The average percent weight gain for animals in the control group was 27.91. Average weight gain for rats administered with 50 mg/kg, 300 mg/kg, and 2000 mg/kg doses of the extract was 10.61, 8.54, and 16.13%, respectively. The extract appeared to have no negative impact on the body weight of the rats, which is why its food and water consumption was not significantly changed. Food and water usage is indicative of normal animal metabolism [36], and this presupposes that the extract of *Faurea speciosa* did not alter rat metabolism. In the 14-day period of acute toxicity evaluation, rats given *Faurea speciosa* extract at dose 50, 300, and 2000 mg/kg recorded no incidence of mortality and all rats did not produce any symptom of toxicity. Therefore, the average acute lethal dose value (LD_50_) is estimated to be more than 2000 mg/kg [27]. The liver is an essential organ involved in metabolism. In the event of liver damage, there will be a rise in transaminase activity, and this activity will also rise when the damage increases [37]. In addition, a number of enzymes typically found in the cytosol are released into the bloodstream when the liver cell membrane is injured. A useful tool for evaluating liver functions is the measurement of activities of the serum marker enzymes ALT, AST, and ALP and serum total bilirubin [38]. Serum markers studied in this work, total bilirubin, indirect bilirubin, direct bilirubin, total protein, ALP, ALT, and AST, exhibited no significant changes relative to the control group (Figure 1). This therefore suggests that the ethanolic extract of *Faurea speciosa* leaf and twig was not hepatotoxic in rats. The kidney is also a key organ that helps in maintaining a stable body state. It excretes metabolic waste products, including drugs and their metabolites. Nonetheless, exposure of the kidney to some of these toxic substances may damage the renal tubules [39]. Notwithstanding their limitations, high serum creatinine, blood urea nitrogen, and low urine output are currently used in diagnosis of acute renal injury [40]. Urea is the metabolic product of protein catabolism, and increase in serum urea might hamper the kidney function if it is not controlled accordingly [41]. In this present study, there was no significant difference in the level of serum creatinine in the extract-treated group compared to that of the control group. However, there were elevated levels of urea in the 50 and 2000 mg/kg bw treated groups (Figure 2). Elevated urea levels may be attributed to the age of the animals or as a result of the diet composition of the rat feed. While urea may not be a major toxin in renal failure, as indicated by Bakir et al. [42], further studies are needed to firmly establish the reason for this occurrence.

## 5. Conclusion

This study has confirmed that *Faurea speciosa* possesses antiplasmodial activity in *in vivo* models. In addition, the extract possesses moderate anti-inflammatory activities. However, the extract's antioxidant ability is average. At concentrations above 2000 mg/kg, the extract appeared to be safe. However, elevated urea levels may be a concern and need further investigation. The results obtained in this study therefore support the traditional use of *Faurea speciosa* for managing malaria and malaria-like conditions. Isolation of the active principles can provide compounds that could be useful for drug discovery.

## Figures and Tables

**Table 1:** Phytochemical screening of the ethanolic extract of *Faurea speciosa*.

Phytochemical	Results
Tannins	+
Flavonoids	+
Saponins	+
Glycosides	+
Alkaloids	−
Coumarins	+
Triterpenoids	−
Sterols	−

+ indicates that the phytochemical is present; − indicates that the phytochemical was not detected under experimental conditions.

**Table 2:** Suppressive test: effect of ethanolic extract of *Faurea speciosa* on parasitemia and mouse mean survival time.

Treatment	Dose (mg/kg)	% parasitemia	% suppression	Mean survival time (days)
Vehicle	NC	93.72 ± 0.38	—	8.80 ± 1.46
Extracts	100	77.27 ± 7.02^a4^	17.55	14.00 ± 2.38^a4^
	200	58.97 ± 4.23^a4^	37.08	15.80 ± 3.02^a4^
	400	31.27 ± 8.80^a4^	66.63	21.80 ± 1.50^a4^
Artesunate	10	6.40 ± 0.83^a4^	93.17	29.20 ± 0.58^a4^

Values are presented as mean ± SEM, *n* = 3. NC = vehicle-treated group. ^4^Values are significantly different at *p* < 0.0001. ^a^Compared to the vehicle-treated group.

**Table 3:** Effect of *Faurea speciosa* extract on body weight and rectal temperature of *P. berghei*-infected mice in the suppressive test.

Treatment	Dose (mg/kg)	Average body weight (g)			Average rectal temperature (°C)		
		Day 0	Day 3	%Δ*W*	Day 0	Day 3	Δ*T*
Saline	0	21.50	21.20	-1.42	37.83	37.40	-0.43
Extracts	100	19.00	19.80	4.21	38.00	37.47	-0.53
	200	19.70	20.70	5.08	38.07	37.83	-0.23
	400	27.50	27.80	1.09	38.17	37.97	-0.20
Artesunate	10	24.90	25.90	4.02	38.13	38.13	0.00

%Δ*W*: difference in body weight between day 0 and day 3 expressed as a percentage; Δ*T*: difference in rectal temperature between day 0 and day 3.

**Table 4:** Curative test: effect of ethanolic extract of *Faurea speciosa* on parasitemia and mouse mean survival time.

Dose (mg/kg)	Curative			
	% parasitemia		% suppression	MST
	Day 3	Day 7		
NC	63.17 ± 6.77	77.14 ± 9.60	—	9.40 ± 1.31
100	76.58 ± 10.23	41.95 ± 13.13^ab4^	45.62	13.00 ± 1.25^a4^
200	54.83 ± 5.41	22.79 ± 2.24^ab4^	70.46	16.20 ± 2.12^a4^
400	43.05 ± 11.55	21.83 ± 7.87^ab4^	71.70	20.00 ± 1.31^a4^
PC	62.71 ± 5.682	5.33 ± 0.31^ab4^	93.09	28.00 ± 0.20^a4^

Values are presented as mean ± SEM, *n* = 3. NC = vehicle-treated group; PC = artesunate. ^4^Values are significantly different at *p* < 0.0001. ^b^Compared to day 3. ^a^Compared to the vehicle-treated group.

**Table 5:** Effect of *Faurea speciosa* extract on body weight and rectal temperature of *P. berghei*-infected mice in the curative test.

Treatment	Dose (mg/kg)	Body weight (g)			Temperature (°C)		
		Day 3	Day 7	%Δ*W*	Day 3	Day 7	Δ*T*
Saline	0	26.63	25.87	-2.88	37.63	37.80	0.17
Extracts	100	27.83	29.20	4.91	37.30	37.20	-0.10
	200	24.20	24.53	1.38	37.7	37.37	-0.33
	400	22.63	22.87	1.03	37.83	37.60	-0.23
Artesunate	10	23.50	23.87	1.56	38.13	37.93	-0.20

%Δ*W*: difference in body weight between day 0 and day 3 expressed as a percentage; Δ*T*: difference in rectal temperature between day 0 and day 3.

**Table 6:** Prophylactic test: effect of ethanolic extract of *Faurea speciosa* on parasitemia and mouse mean survival time.

Dose (mg/kg)	% parasitemia	% suppression	MST (days)
NC	61.14 ± 13.55	—	10.20 ± 1.31
100	31.88 ± 5.72^a4^	47.86	15.00 ± 1.15^a4^
200	31.90 ± 8.80^a4^	47.82	17.80 ± 2.02^a4^
400	26.33 ± 2.76^a4^	56.93	18.20 ± 2.50^a4^
PC	9.90 ± 1.30^a4^	83.82	29.20 ± 0.18^a4^

Values are presented as mean ± SEM, *n* = 3. NC = vehicle-treated group; PC = mefloquine. ^4^Values are significantly different at *p* < 0.0001. ^a^Compared to the vehicle-treated group.

**Table 7:** Effect of *Faurea speciosa* extract on body weight and rectal temperature of *P. berghei*-infected mice in the prophylactic test.

Treatment	Dose (mg/kg)	Average body weight (g)			Average rectal temperature (°C)		
		Day 3	Day 7	%Δ*W*	Day 3	Day 7	Δ*T*
Saline	0	25.37	23.63	-6.83	37.70	37.47	-0.23
Extracts	100	27.47	27.13	-1.21	38.43	38.20	-0.23
	200	26.93	27.00	0.25	38.13	38.30	0.17
	400	25.77	25.97	0.78	37.80	38.13	0.33
Mefloquine	10	27.63	27.67	0.48	38.23	38.03	-0.20

%Δ*W*: difference in body weight between day 0 and day 3 expressed as a percentage; Δ*T*: difference in rectal temperature between day 0 and day 3.

**Table 8:** Antioxidant activity: total antioxidant capacity and DPPH scavenging effect of the ethanolic extract of *Faurea speciosa*.

Extract/standard drug	DPPH scavenging activity (IC_50_ in *μ*g/mL)	Total antioxidant capacity (mg AAE/g)
*F. speciosa*	499.4 ± 5.8	65.4 ± 0.5
Ascorbic acid	16.3 ± 0.5	NA

Results expressed as mean ± SEM of 3 replicate experiments. NA: not applicable (test was not performed).

**Table 9:** Anti-inflammatory activity of the ethanolic extract of *Faurea speciosa*.

Extract or standard drug (mg/kg)	% decrease in edema
10	41.01 ± 0.15
30	49.96 ± 0.42
100	55.50 ± 0.78
Diclofenac (0.3 mg/kg)	47.61 ± 0.54
Dexamethasone (0.3 mg/kg)	60.03 ± 0.54

Data presented as mean ± SD; *n* = 5.

**Table 10:** Acute toxicity: effect of ethanolic extract of *Faurea speciosa* on body weight of rats.

Dose (mg/kg)	% increase in body weight		
	Days 1-7	Days 1-14	Days 7-14
NC	15.81 ± 2.18	27.91 ± 2.76	10.46 ± 2.05
50	7.08 ± 2.40	10.61 ± 1.82	3.32 ± 1.02
300	3.72 ± 1.50	8.54 ± 0.96	4.67 ± 1.16
2000	10.00 ± 4.31	16.13 ± 4.72	5.59 ± 2.45

Days 1-7 and days 1-14 calculated relative to day 1; days 7-14 calculated relative to day 7.

## Data Availability

All data generated or analyzed during this study are included in this published article.
